# Development and validation of a prognostic nomogram for predicting early recurrence after curative resection of stage II/III gastric cancer

**DOI:** 10.1186/s12957-019-1750-1

**Published:** 2019-12-19

**Authors:** Min Ma, Haifan Xiao, Liang Li, Xianli Yin, Huijun Zhou, Hu Quan, Yongzhong Ouyang, Gang Huang, Xiaorong Li, Hua Xiao

**Affiliations:** 1 Postdoctoral Research Station of Clinical Medicine, The Third Xiangya Hospital of Central South University, Changsha, 410013 China; 2 Department of Gastrointestinal Surgery, The Third Xiangya Hospital of Central South University, Changsha, 410013 China; 3 Department of Cancer Prevention and Control, Hunan Cancer Hospital and the Affiliated Cancer Hospital of Xiangya School of Medicine, Central South University, Changsha, 410013 China; 4 Clinical school of medicine, University of South China, Hengyang, 421000 China; 5 Department of Gastroenterology and Urology, Hunan Cancer Hospital and the Affiliated Cancer Hospital of Xiangya School of Medicine, Central South University, Changsha, 410013 China; 6 Department of Gastroduodenal and Pancreatic Surgery, Hunan Cancer Hospital and the Affiliated Cancer Hospital of Xiangya School of Medicine, Central South University, 283 Tongzipo Road, Changsha, 410013 China

**Keywords:** Nomogram, Gastric cancer, Gastrectomy, Early recurrence, Validation

## Abstract

**Background:**

The biological behavior of early recurrence is more invasive and the prognosis is worse in gastric cancer (GC). The risk of early recurrence (ER) for GC in stage II/III has not been reported of which the majority of GC patients are in China. Therefore, it is necessary to analyze the ER of gastric cancer in stage II/III.

**Methods:**

The medical records of 1511 consecutive stage II/III GC patients who received resections were retrospectively reviewed. They were randomly classified into either a development or validation group at a ratio of 7:3. The nomogram was constructed based on prognostic factors using logistic regression analysis and was validated by bootstrap resampling and validation dataset, respectively. Concordance index (C-index) values and calibration curves were used to evaluate the predictive accuracy and discriminatory capability.

**Results:**

Three hundred eleven patients experienced ER, accounting for 20.58% of the GC patients investigated. Multivariate logistic regression analysis identified tumors located at upper, middle third, or mixed, a positive lymph node ratio ≥ 0.335, pTNM stage III, lymphocyte count < 1.5 × 10^9^/L, postoperative infection complications and adjuvant chemotherapy < 6 cycles were all independent predictors for ER after curative resection of stage II/III GC. The C-index value obtained for the model was 0.780 (95% CI, 0.747–0.813), and the calibration curves of validation group yielded a C-index value of 0.739 (95% CI, 0.684–0.794), suggesting the practicability of the model.

**Conclusions:**

The nomogram which was developed for predicting ER of stage II/III GC after surgery had good accuracy and was verified through both internal and external validation. The nomogram established can assist clinicians in determining the optimal therapy strategies in counseling, adjuvant treatments, and subsequent follow-up planning.

## Introduction

Gastric cancer (GC) is the fifth most commonly diagnosed malignancy and ranks third in cancer-caused death worldwide [1]. To date, curative resection is the only possible curative treatment [1]. Unfortunately, most of the patients in China and Western countries are diagnosed at an advanced stage [2]. For these individuals, prognosis remains dismal even after radical resection, with about 20% of tumor recurrence occurring within 1 year of the initial surgery [3, 4]. It has been well established that early recurrence (ER) after operation leads to occurrence of various types of cancer, including GC, intrahepatic cholangiocarcinoma, and pancreatic ductal adenocarcinoma [4–6]. In order to help guide adjuvant treatment and follow-up decisions, and thus to improve long-term outcomes, it is necessary to have detailed knowledge about the risk factors for ER and to identify those individuals with GC at high risk after radical resection.

It is well documented that using a nomogram can accurately predict the association between significant factors and estimated outcomes by creating an intuitive graph, based on the digital multiple relationships in a regression mode [7–9]. Several studies have investigated the independent risk factors for ER of GC, these studies usually involved only a limited cohort of patients and focused solely on early GC or considered early and advanced GC together [4, 9–11]. In fact, the prognosis of stage II/III GC differs significantly from stage I or IV disease. Therefore, in this retrospective study, for the first time, we developed and externally validated a novel nomogram to predict ER of stage II/III GC following curative resection using the database obtained from the two main tertiary cancer treatment hospitals in China. The aim of this study is to find out the risk factors of ER in stage II/III GC, and rather not we hope targeted measures can be taken to prevent and reduce the ER of stage II/III GC.

## Methods

### Design and patients

**Fig. 1:**
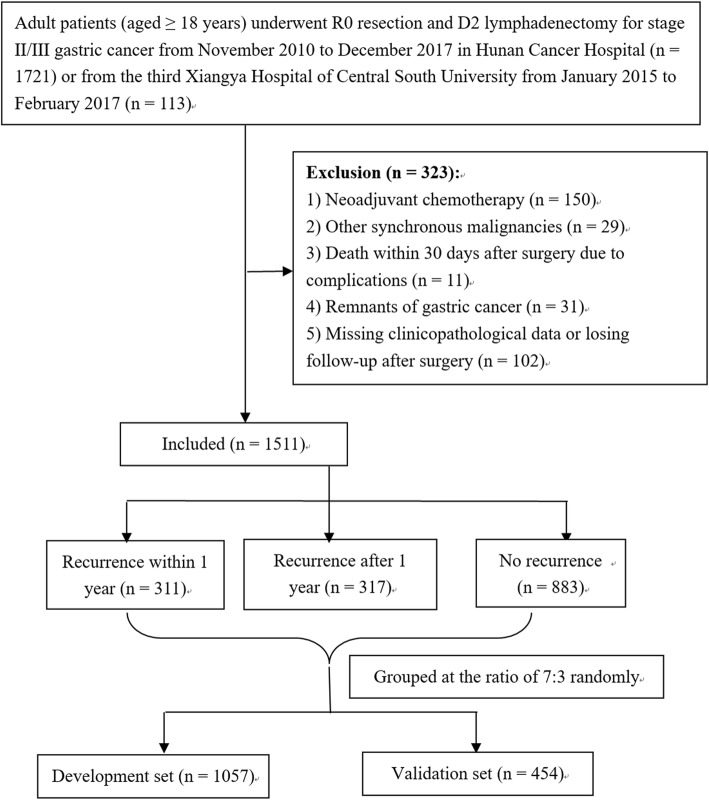
The exclusion criteria and a flow diagram of the investigation

We reviewed retrospectively the medical records of all GC patients who underwent surgery in the Hunan Cancer Hospital between November 2010 and December 2017 and those in the Third Xiangya Hospital of Central South University between January 2015 and February 2017. Adult patients (≥ 18 years old) with pathologically confirmed stage II/III gastric adenocarcinoma who underwent curative resection (R0 resection and D2/D2+ lymphadenectomy) were eligible for inclusion in the study. The ethics committee of the Hunan Cancer Hospital and the Third Xiangya Hospital of Central South University approved the protocols, which complied with the standards of the Declaration of Helsinki for experiments on humans. Every enrolled patient provided written signed consent forms permitting surgery and analysis of their clinical data. To test the general applicability of the model, patients were randomly assigned to a development group (*n* = 1057) or validation group (*n* = 454) at a ratio of 7:3. Figure 1 shows the exclusion criteria and a flow diagram of the investigation.

### Perioperative management and follow-up

Expert surgeons with major experience in curative gastrectomy and D2 lymphadenectomy carried out the operations. Lymphadenectomy and digestive tract reconstruction followed the Japanese GC surgical guidelines [12]. The TNM stage was classified based on the American Joint Committee on Cancer TNM Staging System (8th edition) [13]. Our previous studies have described the main surgical procedures, perioperative management, and follow-ups [3, 14]. Briefly, postoperative complications were identified within 30 days of surgery and classified using Clavien-Dindo criteria [15]. Based on the patients’ wishes and their physical condition, fluorouracil- and platinum-based regimens (generally 3-week cycles of capecitabine/S-1 and oxaliplatin) as adjuvant chemotherapy was recommended for those with stage II/III GC [16, 17], usually started about 3 to 4 weeks after surgery.

Every patient was followed-up either with an outpatient visit or by telephone 1 month after their initial surgery, every 3 months during the first 2 years, at 6-month intervals between years 3 and 5, and thereafter on a yearly basis. Routine assessments included physical examinations, laboratory tests (including routine blood test, carcinoembryonic antigen, and carbohydrate antigen 199), an ultrasonography, and/or computed tomography (CT) scan performed every 6 months during the 5 years following surgery and endoscopy was performed every 2 years. The latest follow-up date was obtained during December 2018.

### Evaluation

Clinicopathological variables that included demographic, operative details, pathological, and follow-up findings were obtained from patient medical records. Routine laboratory measurements including lymphocyte counts, hemoglobin, and serum albumin levels were measured in each patient within 7 days of their operation. As previously reported, the prognostic nutritional index (PNI) was evaluated using the following equation: PNI = serum albumin level (g/L) + 0.005 × the peripheral blood (per mm^3^) total lymphocyte count [18]. The cut-off values were set at 1.5 × 10^9^/L and 45 for the lymphocyte count and PNI values, respectively [18, 19]. The positive lymph node ratio (PLNR) was calculated as the total pathological metastasis lymph node numbers/the total retrieved lymph node numbers. A receiver operating characteristic (ROC) curve was generated to estimate the optimal cut-off value for PLNR as a risk factor for ER. Whereas for other commonly used variables such as age, body mass index (BMI), albumin levels or anemia, standard clinical, or widely accepted thresholds were used.

The diagnosis of recurrence was based on radiologic findings and/or suspicious lesions after examination of biopsy specimens. ER was diagnosed as recurrence within 1 year of the initial operation for GC, as reported in previous studies [4, 5].

### Statistical analysis

Student’s *t* test, *χ*2 test, or Fisher’s exact test was used for statistical analysis of continuous variables or categorical variables. All variables were entered into the logistic regression model by the backwards step-down process of the Akaike information standard stop rules. The nomogram prediction model was constructed based on the data revealed by multivariate logistic regression analysis, including bootstrap self-sample verification and external verification. At the same time, 1000 pilot resampling of the development data set was used to implement self-sample verification of the program. The source data was divided into two groups: a larger set was used to develop the model, and a smaller set was used for external verification of the built model. The predicted values of the model were evaluated based on the rank consistency index value (C-index) on a scale from 0 to 1 and a 95% confidence interval (CI), and the area under the curve (AUC) of the ROC curves. A C-index value > 0.70 indicates that the model was advantageous for differentiation. The nomogram was calibrated by comparing the observed and predicted rates of the ER. All statistical analysis and data processing were performed using R software (Ver. 3.5.1, R Foundation for Statistical Computing). And all tests were bilateral and *P* < 0.05 were considered significant.

## Results

### Clinical, pathological characteristics, and bivariate analysis

Stage II/III GC patients (1511 in total) were enrolled of whom 1057 were distributed to the nomogram development set and the remaining 454 into the external validation set. In the nomogram development set, there were 700 men (66.20%) and 357 women (33.80%). The median age in years at diagnosis was 55.71 (range 19–90 years). A total of 216 (20.40%) patients presented with ER. In the external validation set, there were 302 men (66.50%) and 152 women (33.50%). At diagnosis, the median age was 55.87 (range 24–82 years). A total of 95 (20.90%) patients experienced ER. The bivariate analyses of the development set showed that age, BMI, tumor diameter, tumor location, lymphocyte count, PNI, gastrectomy extent, PLNR, adjuvant chemotherapy, perioperative blood transfusion, postoperative infection complications, pT stage, pN stage, and pTNM stage were significantly associated with ER (*P* < 0.05 for all, Table 1).

**Table 1:** Clinicopathological characteristics of all the patients (*n* = 1511)

Variables ^a^	Nomogram develop set (*n* = 1057)			External validation set (*n* = 454)		
	Early recurrence		*P* value	Early recurrence		*P* value
	Yes	No		Yes	No	
	(*n* = 216)	(*n* = 841)		(*n* = 95)	(*n* = 359)	
Age (years)	57.28 ± 11.70	55.31 ± 10.75	0.018	56.35 ± 11.98	55.74 ± 10.20	0.618
Sex			0.741			0.770
Female	75 (7.10%)	282 (26.88%)		33 (7.27%)	119 (26.21%)	
Male	141 (13.34%)	559 (52.89%)		62 (13.66%)	240 (52.86%)	
Body mass index (kg/m^2^)	21.14 ± 2.92	21.82 ± 2.90	0.002	21.23 ± 2.89	22.00 ± 2.97	0.025
Any comorbidities			0.208			0.923
No	158 (14.95%)	578 (54.68%)		67 (14.76%)	255 (56.17%)	
Yes	58 (5.49%)	263 (24.88%)		28 (6.17%)	104 (22.91%)	
ASA score			0.097			0.770
1	29 (2.74%)	129 (12.20%)		17 (3.74%)	70 (15.42%)	
2	152 (14.38%)	619 (58.56%)		66 (14.54%)	247 (54.41%)	
3	35 (3.31%)	89 (8.42%)		11 (2.42%)	41 (9.03%)	
4	0 (0%)	4 (0.38%)		1 (0.22%)	1 (0.22%)	
Hemoglobin (g/L)	112.87 ± 26.06	117.61 ± 25.07	0.014	114.61 ± 27.02	119.03 ± 25.02	0.132
Albumin level (g/L)	37.27 ± 5.14	38.08 ± 4.58	0.024	37.74 ± 4.74	37.95 ± 4.40	0.684
Lymphocyte count (×10^9^/L)	1.62 ± 0.61	1.77 ± 0.66	0.002	1.66 ± 0.67	1.76 ± 0.60	0.153
Prognostic nutritional index	45.35 ± 6.53	46.94 ± 6.07	0.001	46.01 ± 6.39	46.73 ± 5.66	0.286
Tumor diameter (cm)	5.26 ± 2.25	4.48 ± 2.00	< 0.001	5.10 ± 2.18	4.52 ± 2.00	0.014
Tumor location			< 0.001			0.002
Upper	27 (2.55%)	74 (7.00%)		12 (12.64%)	32 (7.05%)	
Middle	60 (5.68%)	185 (17.50%)		30 (6.61%)	81 (17.84%)	
Lower	112 (10.60%)	561 (53.07%)		43 (9.47%)	233 (51.32%)	
Mixed	17 (1.61%)	21 (1.99%)		10 (2.20%)	13 (2.86%)	
Gastrectomy extent			< 0.001			< 0.001
Sub-total	124 (11.73%)	642 (60.74%)		53 (11.67%)	268 (59.03%)	
Total	92 (8.70%)	199 (18.83%)		42 (9.25%)	91 (20.04%)	
Pathological type			0.131			0.541
Differentiated	15(1.42%)	87 (8.23%)		9 (1.98%)	42 (9.25%)	
Undifferentiated	201 (19.02%)	754 (71.33%)		86 (18.94%)	317 (69.82%)	
Positive lymph node ratio	0.46 ± 0.29	0.23 ± 0.24	< 0.001	0.43 ± 0.31	0.23 ± 0.24	< 0.001
Adjuvant chemotherapy			< 0.001			< 0.001
≥ 6 cycles	45 (4.26%)	286 (27.06%)		15 (3.30%)	123 (27.09%)	
<6 cycles	171 (16.18%)	555 (52.51%)		80 (17.62%)	236 (51.98%)	
Peri-operative blood transfusion			< 0.001			0.025
No	142 (13.43%)	670 (63.39%)		66 (14.54%)	288 (63.44%)	
Yes	74 (7.00%)	171 (16.18%)		29 (6.39%)	71 (15.64%)	
Post-operative infection complications			< 0.001			0.660
No	189 (17.88%)	798 (75.50%)		88 (19.33%)	337 (74.23%)	
Yes	27 (2.55%)	43 (4.07%)		7 (1.54%)	22 (4.85%)	
pT stage			< 0.001			0.226
T1	1 (0.09%)	19 (1.80%)		1 (0.22%)	7 (1.54%)	
T2	5 (0.47%)	89 (8.42%)		4 (0.88%)	38 (8.37%)	
T3	12 (1.14%)	70 (6.62%)		6 (1.32%)	26 (5.73%)	
T4	198 (18.73%)	663 (62.72%)		84 (18.50%)	288 (63.44%)	
pN stage			< 0.001			< 0.001
N0	12 (1.14%)	203 (19.21%)		11 (2.42%)	96 (21.15%)	
N1	28 (2.65%)	178 (16.84%)		13 (2.86%)	71 (15.64%)	
N2	36 (3.41%)	229 (21.67%)		20 (4.41%)	94 (20.70%)	
N3	140 (13.25%)	231 (21.85%)		51 (11.23%)	98 (21.59%)	
pTNM stage			< 0.001			< 0.001
II	14 (1.32%)	294 (27.81%)		14 (3.08%)	137 (30.18%)	
III	202 (19.11%)	547 (51.75%)		81 (17.84%)	222 (48.90%)	

^a^Continuous were present as mean ± SD and or categorical data were presented as *n*

### Prognostic nomogram for early recurrence

**Fig. 2:**
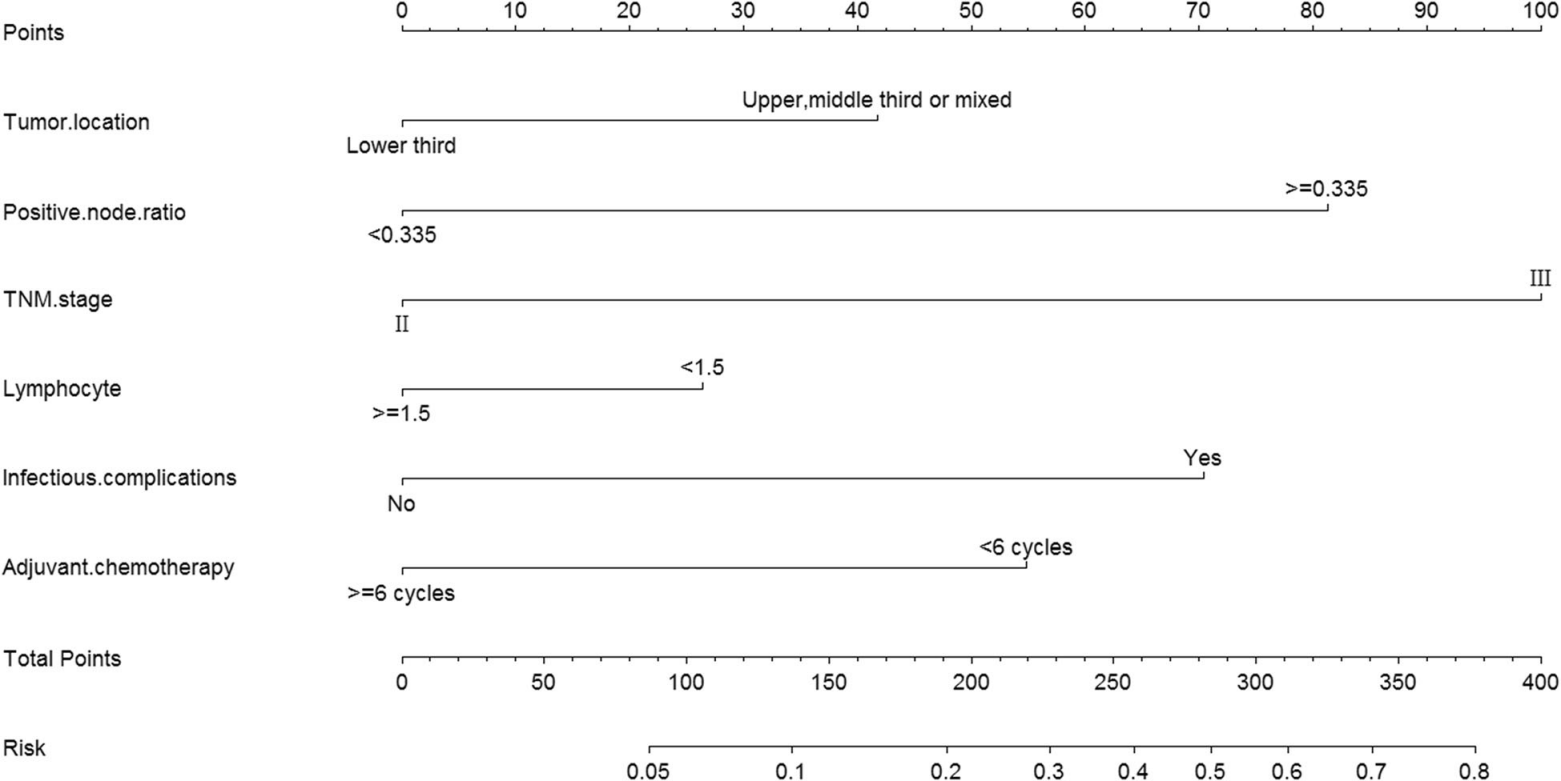
The prognostic nomogram of early recurrence

Multivariate logistic regression analysis showed that tumors located at upper, middle third, or mixed, PLNR ≥ 0.335, pTNM stage III, lymphocyte count < 1.5 × 10^9^/L, postoperative infection complications and adjuvant chemotherapy of < 6 cycles were independent risk factors for ER (Table 2). Based on 6 prognostic factors, a prognostic nomogram of ER was constructed (Fig. 2). The outcomes showed that pTNM stage III was the largest risk factor for ER. Other factors, including PLNR ≥ 0.335, postoperative infection complications, adjuvant chemotherapy < 6 cycles, tumor located at upper, middle third, or mixed, and lymphocyte count < 1.5 × 10^9^/L contributed to ER in descending order of importance.

**Table 2:** Multivariate analyses of risk factors for early recurrence in the development set (logistic regression model)

Variable	Odds ratio	95% Confidence interval	*P* value	Score^#^
Tumor location				
Lower third	1			0
Upper, middle third or mixed	1.87	1.34–2.60	< 0.001	41.5
Positive lymph node ratio				
< 0.335	1			0
≥ 0.335	3.37	2.37–4.78	< 0.001	81.0
pTNM stage				
II	1			0
III	4.45	2.44–8.09	< 0.001	100.0
Lymphocyte count				
≥ 1.5 × 10^9^/L	1			0
< 1.5 × 10^9^/L	1.48	0.48–0.94	0.021	26.0
Postoperative infection complications				
No	1			0
Yes	2.85	1.61–5.07	< 0.001	70.0
Adjuvant chemotherapy				
≥ 6 cycles	1			0
< 6 cycles	2.27	1.54–3.30	< 0.001	55.0

^#^Score was derived from the nomogram plot (Fig. 2)

### Calibration and nomogram validation

**Fig. 3:**
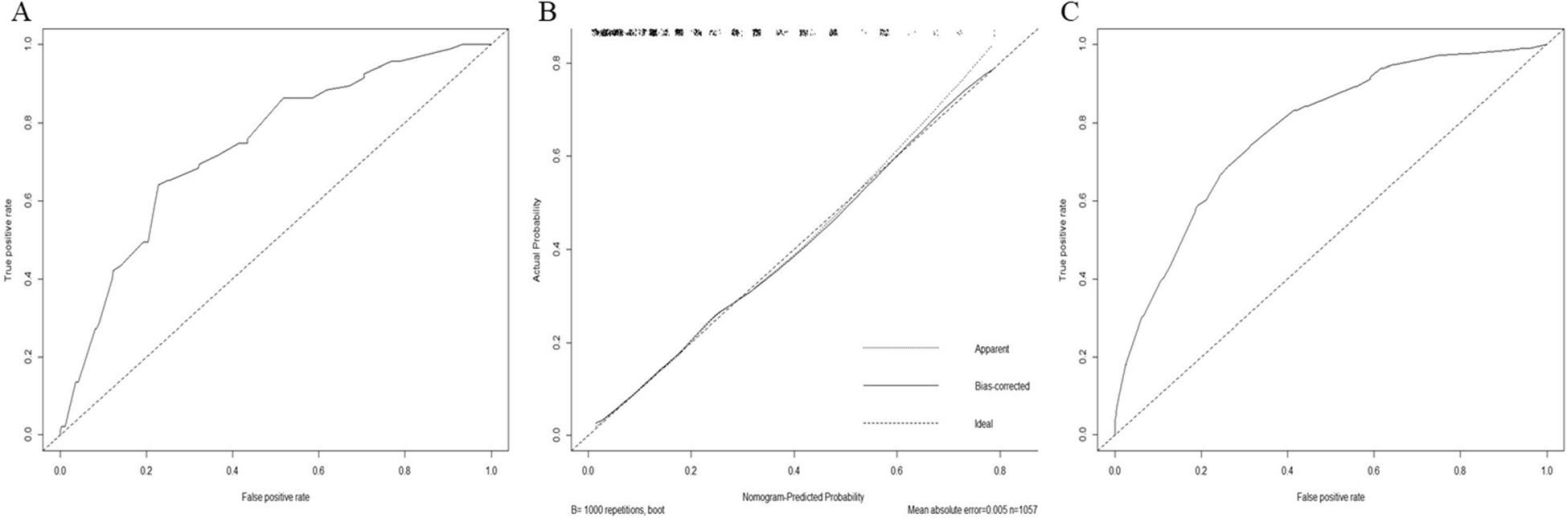
**a** Internal validation of the nomogram model yielded a C-index value of 0.780 (95% CI, 0.747–0.813), which was identical to the area under curve of the receiver operating characteristic curves. **b** The internal calibration curve showed optimal agreement between nomogram predictions and actual observations. **c** Data for the validation group were used for the external validation of the nomogram model, which yielded a C-index value of 0.739 (95% CI, 0.684–0.794)

Internal validation of the nomogram model yielded a C-index value of 0.780 (95% CI, 0.747–0.813), which was identical to the AUC of the ROC plot (Fig. 3a). The internal calibration curve showed optimal agreement between nomogram predictions and actual observations (Fig. 3b). Data for the validation group (*n* = 454) were used for the external validation of the nomogram model, which yielded a C-index value of 0.739 (95% CI 0.684–0.794), which was similar to the index of the development group (Fig. 3c).

### Importance of adjuvant chemotherapy at GC stage III

After multivariate regression analysis, we analyzed the role of postoperative adjuvant chemotherapy. The results showed an ER rate of 16.3% in patients who completed 6 cycles of adjuvant chemotherapy, which was significantly lower than that of 31.9% in patients who received < 6 cycles of chemotherapy. Further stratified analyses revealed that regardless of tumor location, lymphocyte count, PLNR, and postoperative infection complications, completion of 6 cycles of chemotherapy significantly reduced the rate of ER in stage III GC patients (Table 3).

**Table 3:** The early recurrence rate of patients with stage III gastric cancer

Variable	Adjuvant chemotherapy ≥ 6 cycles	Adjuvant chemotherapy < 6 cycles	*P*
Tumor location			
Lower third	27/206 (13.1%)	114/434 (26.3%)	< 0.001
Upper, middle third, or mixed	28/132 (21.2%)	114/280 (40.7%)	< 0.001
Positive lymph node ratio			
< 0.335	15/172 (8.7%)	74/371 (19.9%)	0.001
≥ 0.335	40/166 (24.1%)	154/343 (44.9%)	< 0.001
Lymphocyte count			
≥ 1.5 × 10^9^/L	31/214 (14.5%)	124/439 (28.2%)	< 0.001
< 1.5 × 10^9^/L	24/124 (19.4%)	104/275 (37.8%)	< 0.001
Postoperative infection complications			
No	51/318 (16.0%)	202/661 (30.6%)	< 0.001
Yes	4/20 (20.0%)	26/53 (49.1%)	0.024

## Discussion

At present, the prognosis of stage II/III GC remains poor, mainly due to its recurrence and metastasis [1]. The recurrence of GC presents with a certain time specificity: the probability of recurrence gradually increased, reached a high point, and then gradually decreased [20]. Moreover, patients who experienced ER had significantly shorter survival times compared with those suffered late recurrence [11, 21]. Therefore, it is essential to clarify the risk factors of ER following an operation, to provide more rigorous follow-up and more radical treatment, and as a result delay recurrence and ultimately improve the long-term outcomes for patients.

Although several studies have investigated the risk factors for ER of GC, a clear definition of ER was lacking, which varied from 12 to 36 months [4, 9–11]. In the present study, we choose 1-year recurrence as the ER, for the reason that 1 year was the optimal threshold based on the differences in post-recurrence survival in GC patients reported by Xu et al. [4]. The result was echoed by Groot VP et al. [5], who argued that a recurrence-free interval of 12 months was also the optimal threshold for differentiating between early and late recurrence for resected pancreatic ductal adenocarcinoma based on subsequent prognosis. In addition, other research has focused on the ER of early GC, but the prognosis and recurrence of locally advanced GC were different from those in early GC patients. Our study is the first to develop and validate a nomogram to predict ER of patients with stage II/III GC following radical gastrectomy, using patients’ data from two high-volume tertiary hospitals in China. As a result, we found 311 patients with GC at stage II/III had ER, which accounted for 20.58% of the enrolled patients, comparable findings (15.79%) to those reported by Xu et al. [4].

The nomogram, also known as the alignment diagram, is based on multi-factor regression analysis, which integrates multiple predictive indicators and then uses scaled segments to follow a certain percentage. The nomogram transforms complex regression equations into visualized graphs, making the results of the predictive model more readable and easy to evaluate patients’ conditions. It is precisely because of the intuitive and easy to understand characteristics of the nomogram that it has gradually gained more attention and applications in medical research and clinical practice [22]. In our study, the risk factors for ER were linearized, simple, and easy to interpret. The C-indexes were 0.780 and 0.739 in the experimental and verification group, respectively. And the coincidence degree between the actual curve and the predicted curve was in accord, indicating that our model had strong prediction ability and could be usefully applied in clinical practice.

As shown in Fig. 2, pTNM stage III was the strongest predictor for ER. The pTNM stage is evaluated according to metastasis of lymph nodes, distant metastasis, and the depth of tumor invasion. It comprehensively reflects the progress of GC and the biological behavior of the tumor(s). Thus, it was not surprising that pTNM stage III was the most important reference factor for ER prediction. The next most important factor that affects ER of GC was PLNR. As is well known, GC is prone to lymph node metastasis (LNM), even in early GC [23, 24]. Studies have shown that LNM of GC is one of the essential factors affecting the prognosis. In the present study, a PLNR ≥ 0.335 was found to be an independent risk factor for ER in patients with stage II/III GC, which was echoed by Komatsu et al [25], who concluded that patients with a PLNR value ≥ 0.4 had a higher rate of node recurrence than patients with a PLNR value < 0.4 with pN3 GC. Thus, early lymph node recurrence might explain the relationship between higher PLNR and ER, but the exact type of recurrence in our study was not investigated and further prospective studies are needed.

Postoperative infection complication was also identified as one of the causes of ER of GC. Possible explanations included immune suppression caused by infection and delayed adjuvant chemotherapy [14, 26]. Our previous study has demonstrated that overweight (BMI ≥ 25.0 kg/m^2^) was an independent risk factor for postoperative infection [14]. Whereas the harvested lymph node may decrease in overweight GC patients (the mean harvested lymph node was 19.5 ± 7.2 in patients with BMI ≥ 25.0 kg/m^2^, whereas it was 22.9 ± 8.8 in those with BMI < 25.0 kg/m^2^ in the present study, *P* = 0.014) and the number of harvested lymph node also independently affects the prognosis of GC patients who underwent radical resection. Thus we must bear in mind that the harvested lymph node may be a confounder when investigating the relationship between postoperative infection and ER of GC. Further, propensity score matching analyses that could balance the baseline data may offer statistical power to improve the reliability of our final conclusions.

Adjuvant chemotherapy is recommended as a standard treatment following curative resection of stage II/III GC in guidelines for both Western and Eastern countries [12, 27]. However, it is not uncommon to encounter patients who cannot complete the full course of planned adjuvant chemotherapy for various reasons including economic burden, poor general physical condition, and side effects of chemotherapy. In fact, almost 50% of patients did not complete the allocated postoperative treatment as planned, even in recent prospective large-scale randomized controlled studies [28–30]. Incomplete adjuvant chemotherapy has been identified to independently predict poor outcomes for patients with various types of malignancies [28, 31]. In the present study, adjuvant chemotherapy of < 6 cycles is a risk factor for ER which emphasizes the importance of postoperative adjuvant chemotherapy. In addition, we compared the recurrence-free survivals (RFS) between stages II and III, and thereafter, investigated the influence of adjuvant therapy on RFS both in stage II and III GC. RFS did not differ significantly in stage II patients, but differ significantly in stage III patients, divided by receiving 6 cycles of adjuvant chemotherapy or not (as shown in Additional file 1: Figure S1). The possible explanation was the relatively small number of patients with stage II diseases in this study, which may hamper the statistic power, and further studies with large sample size are needed.

Previous studies had demonstrated that cancers located in the upper third or mixed were associated with significantly poorer prognosis, which was independent of the tumor stage and other clinicopathological variables, and should be acknowledged as an important prognostic factor [31, 32]. In addition, non-antral GC was more commonly associated with deeper invasion, more lymph node metastasis, and lymphatic vessel invasion. Thus, it was not surprising that tumors located at the upper, middle third, or mixed were independent risk factors for ER compared with lower-third GC. A lymphocyte count < 1.5 × 10^9^/L was also found to be associated with ER in stage II/III GC, which might explain the conclusions of our previous study, in which lymphocyte counts < 1.5 × 10^9^/L were found to be an independent risk factor for poorer OS and DFS [18]. Some studies have reported that preoperative enteral nutrition in GC could improve postoperative immune status and reduce postoperative infection [33]. Whether improved nutrition can reduce ER and thus improve long-term outcomes needed further prospective studies for clarification of this idea.

There were a number of limitations in our findings. It was a retrospective study and the predictive factors involved in our nomogram were acquired from routine demographic and laboratory testing. Some other important factors, such as the exact dose and regimens used for chemotherapy, the exact reason for incompleteness of adjuvant chemotherapy and the exact recurrence type were not investigated in the present study, which may serve as a confusing factor in our study. And the sample size for the dataset used for nomogram external validation was relatively small. Thus, further studies with large sample size are needed to verify the usefulness of this model and generalizability of our conclusions.

Although more and more studies have shown neoadjuvant chemoradiotherapy could be performed before radical surgery for advanced gastric cancer in Western countries [34], there are still a small number of patients in Asia, such as Japan, Korea, and China, who have undergone neoadjuvant therapy. In our data, the percentage was just 8.7%. Moreover, considering the pathological diagnosis staging after neoadjuvant is different from patients without neoadjuvant chemotherapy (ypTNM staging). In order to avoid confusion, this study excluded the patients with neoadjuvant chemotherapy, which may affect generalization of the conclusion to some extent.

Notwithstanding these limitations, it is the first study to develop and externally validate a novel nomogram to predict ER of stage II/III GC following curative resection based on a large cohort of patients. Our research should enable doctors to determine accurately the possibility of ER of stage II/III GC and thus adopt different treatment options. From these significant factors for ER in the model, doctors should be encouraged to try their best to reduce the incidence of postoperative infections, persuade patients to complete at least six cycles of adjuvant chemotherapy, and pay more attention to the immune status of patients, especially those with tumor(s) not located at the lower third, and with a higher PLNR.

In conclusion, the present study identified that tumor(s) located at the upper, middle third or mixed, PLNR ≥ 0.335, pTNM stage III, lymphocyte count < 1.5 × 10^9^/L, postoperative infection complications, and adjuvant chemotherapy < 6 cycles were risk factors for ER after curative resection of GC. A nomogram model can be used to predict the possibility of ER in patients with GC at stages II or III. Further investigations will be required to clarify whether immuno-nutrition intervention and completion of adjuvant chemotherapy could reduce the risk of ER, and as a result, improve the prognosis of GC patients.

## Supplementary information

**Additional file 1: Figure S1.** Recurrence-free survivals of gastric cancer patients who underwent radical gastrectomy divided by receiving 6 cycles of adjuvant chemotherapy or not. **A** in stage II patients (*P* = 0.263 by log-rank test). **B** in stage III patients (*P* < 0.001 by log-rank test).

## Data Availability

The database used and/or analyzed during the current study is not publicly available (to maintain privacy) but can be available from the corresponding author on reasonable request.

## Abbreviations

AUC: Area under the curve
CI: Confidence interval
CT: Computed tomography
ER: Early recurrence
GC: Gastric cancer
LNM: Lymph node metastasis
PLNR: Positive lymph node ratio
PNI: Prognostic nutritional index
ROC: Receiver operating characteristic
