# Supplementary figures and images for: Development and validation of a prognostic nomogram for predicting early recurrence after curative resection of stage II/III gastric cancer
Source: World J Surg Oncol. 2019 Dec 19;17:223. doi: 10.1186/s12957-019-1750-1 (PMC6923869; doi:10.1186/s12957-019-1750-1)

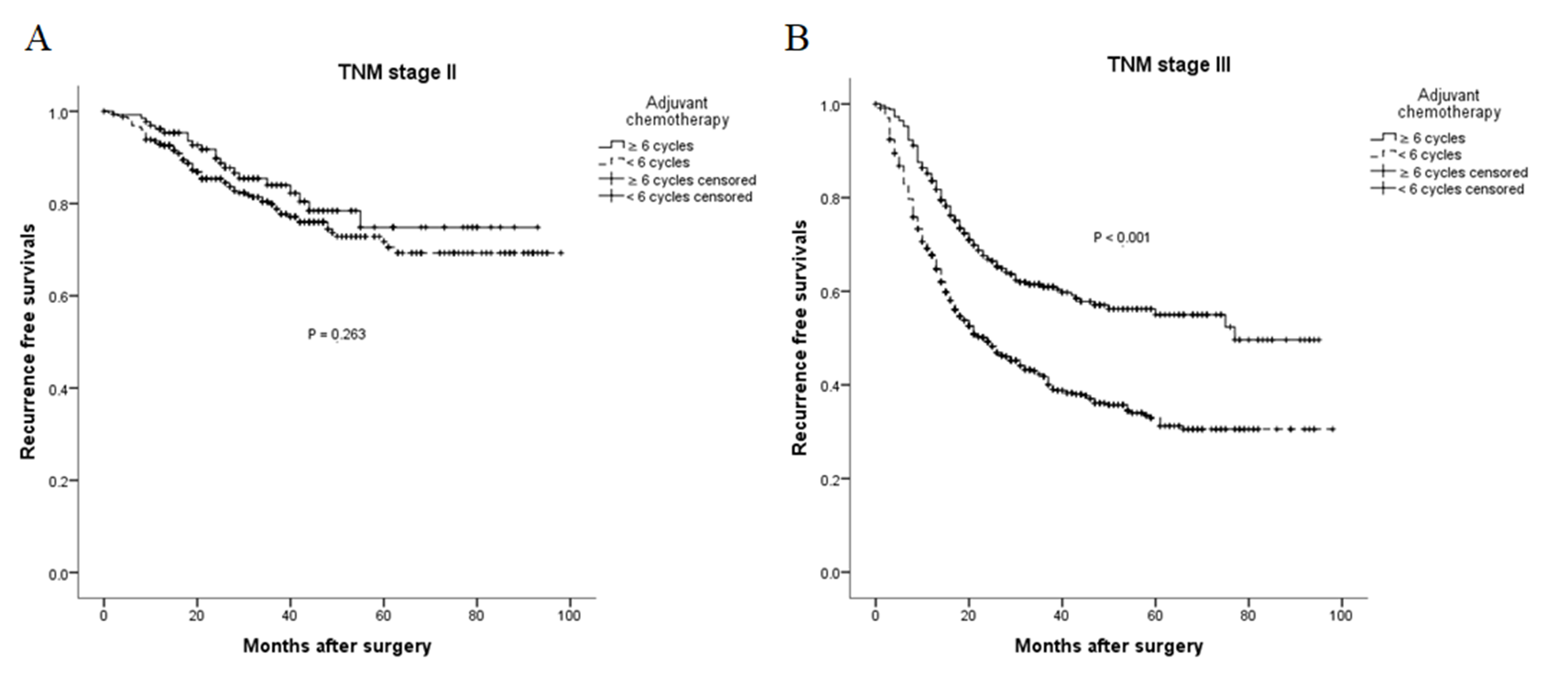

Supplement: Supplementary file 1 — Additional file 1: Figure S1. Recurrence-free survivals of gastric cancer patients who underwent radical gastrectomy divided by receiving 6 cycles of adjuvant chemotherapy or not. A in stage II patients (P = 0.263 by log-rank test). B in stage III patients (P < 0.001 by log-rank test). [file 12957_2019_1750_MOESM1_ESM.tif]
